# Composite Bonding Pre-Treatment with Laser Radiation of 3 µm Wavelength: Comparison with Conventional Laser Sources

**DOI:** 10.3390/ma11071216

**Published:** 2018-07-16

**Authors:** David Blass, Sebastian Nyga, Bernd Jungbluth, Hans-Dieter Hoffmann, Klaus Dilger

**Affiliations:** 1 Institute of Joining and Welding, Technische Universität Braunschweig, Langer Kamp 8, 38106 Braunschweig, Germany; k.dilger@tu-braunschweig.de; 2 Fraunhofer Institute for Laser Technology ILT, Steinbachstr. 15, 52074 Aachen, Germany; sebastian.nyga@ilt.fraunhofer.de (S.N.); bernd.jungbluth@ilt.fraunhofer.de (B.J.); hansdieter.hoffmann@ilt.fraunhofer.de (H.-D.H.)

**Keywords:** laser ablation, CFRP, frequency conversion, surface treatment

## Abstract

To use the full potential of composite parts, e.g., to reduce the structural weight of cars or airplanes, a greater focus is needed on the joining technology. Adhesive bonding is considered favorable, superior joining technology for these parts. Unfortunately, to provide a structural and durable bond, a surface pre-treatment is necessary. Due to its high integration potential in industrial process chains, laser radiation can be a very efficient tool for this purpose. Within the BMBF-funded (German Federal Ministry of Education and Research) project GEWOL, a laser source that emits radiation at 3 µm wavelength (which shows significant advantages in theory) was developed for a sensitive laser-based bonding pre-treatment. Within the presented study, the developed laser source was compared with conventional laser sources emitting radiation at 355 nm, 1064 nm, and 10,600 nm in terms of application for a composite bonding pre-treatment. With the different laser sources, composites were treated, analytically tested, subsequently bonded, and mechanically tested to determine the bonding ability of the treated specimens. The results show a sensitive treatment of the surface with the developed laser source, which resulted in a very effective cleaning, high bonding strengths (over 32 MPa), and a good effectiveness compared with the conventional laser sources.

## 1. Introduction and State of the Art

The main application of composites like carbon fiber reinforced plastics (CFRP) addresses the increase of the eco-efficiency of airplanes and cars by reducing their structural weight, which reduced the fuel/energy consumption. Nevertheless, the final structure typically consists of different parts made out of different materials, which requires the joining of those parts. Therefore, the development of an efficient joining technology for CFRP-parts becomes more and more relevant [1].

Unfortunately, the joining of CFRPs has several challenges. On the one hand, conventional joining technologies show disadvantages and some are not applicable. On the other hand, adhesive bonding of those materials requires more effort compared to the bonding of classical construction materials. Focusing on the disadvantages of conventional joining techniques, riveting is a useful example. Like all mechanical joining processes, it has a significant disadvantage in which the load bearing fibers are cut, which results in a weaker structure and a stress concentration around the rivet. This needs to be leveled by an additional reinforcement correlating with a higher structural weight. Welding as another commonly used joining technique cannot be applied for thermoset composites because the materials cannot be melted. Adhesive bonding, in contrast, allows the joining of almost any kind of material and leads to a favorable stress distribution. Nevertheless, to provide a structural and durable bond, the cohesive performance of the applied adhesive needs to be high and the adhesion on the part’s surfaces needs to be guaranteed. Unfortunately, the adhesion is reduced by the presence of mold-release agent residues and other contaminations and, therefore, the bonding performance of untreated composites is limited [2,3].

As such, thermoset composites require a surface treatment prior to the application of the adhesive to provide a sufficient adhesion on their surfaces. Within industrial processes, this bonding pre-treatment is completed mostly by mechanical processes (grinding or blasting processes) or by the application of so called peel-plies (fabrics that are laminated on top of the structural fibers of the CFRP part prior to curing and torn out after the curing process). Both methods have relatively bad integration potentials in a highly automated process chain (e.g., manual grinding) or show additional disadvantages. Possible disadvantages include the development of dust for conventional blasting processes [4], limitations in terms of the part geometry (low pressure blasting [LPB]) [4], or a limited cleaning effect (e.g., atmosphere plasma) [5].

A method, which provides a high automation potential with reduced wear and a high flexibility, is applied toward laser radiation, which is shown in several fields such as cutting, welding [6], and materials testing [7]. Based on this potential, several investigations about the applicability of different laser sources [8,9,10,11,12,13,14,15,16,17,18] to clean the surface in the context of bonding pre-treatment and characterize the ablation behavior of different CFRP types were made.

**Figure 1:**
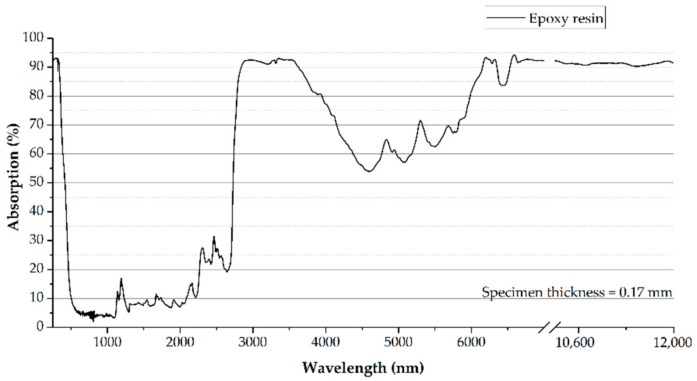
Absorption curve for a typical epoxy resin [18].

In summary, there are several set-ups in which—within a laboratory scale—a sufficient bonding pre-treatment is possible. However, to establish laser radiation as a tool within industrial applications, specific challenges have to be solved. The main obstacle is the high initial investment cost for a laser system. Furthermore, laser systems for the pre-treatment of adherents typically comprise lasers emitting in the ultraviolet (UV) wavelength range [13], e.g., excimer lasers [14,15,16,17]. UV-lasers exhibit severe limitations since the components of the laser are subjected to UV-radiation, which leads to degradation effects. Therefore, UV-lasers require frequent maintenance, which leads to high operational costs. Highly mature lasers with lifetimes spanning over several 10,000 h can be provided at low cost in the near-infrared, e.g., solid state or fiber-lasers at 1064 nm, and in the mid-infrared, e.g., CO_2_-lasers at 10,600 nm [9,13]. The transparency of epoxy (one of the most common resins in CFRP) is typically high in the near-infrared region (see Figure 1) [18]. Resulting, the major part of the irradiated light will be absorbed by the fibers, which consequently removes the resin based on the difference of thermal elongation of fibers and resin. This can lead to delamination effects in the compound material [11,12,18]. Even though the absorption in epoxy is high at 10,600 nm, the low photon energy of CO_2_-laser radiation can lead to thermal ablation and still causes delamination effects. Even if there are processing parameters that allow for a sufficient bonding pre-treatment with fiber/solid state and CO_2_ lasers, the processing window is typically small and, therefore, the required robustness for industrial applications cannot be guaranteed.

To guarantee a robust process, a new approach was chosen within the GEWOL project. Using a laser emitting at 3 µm wavelength, which is highly absorbed by epoxy (see Figure 1) while still having a significant higher photon energy compared with CO_2_ laser radiation, an efficient pre-treatment of adherents will be demonstrated without delamination effects. The laser radiation shall be provided by using a low cost industrial laser at 1064 nm and a subsequent efficient frequency conversion to 3 µm. First implementations of a 3 µm laser and bonding pre-treatment experiments were presented by the authors [8,18,19]. This paper focuses on the comparison of the pre-treatment results of adherents at a 3 µm wavelength with the results at wavelengths emitted by conventional lasers.

## 2. Methodology and Experimental Set-Up

Within the presented examinations, typical aerospace-related CFRP-samples were treated with laser radiation of four different wavelengths. To determine the treatment efficiency of each laser source as well mechanical tests of bonded specimens, analytical tests of the treated surfaces were performed while the surfaces were pre-treated with comparable treatment intensities (aerial energies).

### 2.1. Methodology

To compare the efficiency of a bonding pre-treatment with different laser sources, Kreling [13] established an approach defining an applied aerial energy. In this case, the aerial energy, which is applied to the specimen’s surface, is calculated by multiplying the average amount of laser pulse hits per surface increment with the laser pulse fluence.

Unfortunately, the efficiency of different laser sources is difficult to determine because, for every source, there is a (potentially small) process window, which leads to high bonding strengths. Therefore, the applied aerial energy was chosen to be the judgement parameter since, due to the risk of causing material damage, an efficient treatment correlates with a low aerial energy while guaranteeing a cohesive failure inside the adhesive and no occurrence of material damage (e.g., delaminations).

For comparable material systems as used within this study, the required aerial energies to achieve high lap shear strengths can be stated to be roughly 60 mJ/mm^2^ for UV-laser sources (355 nm) where high bonding strengths could be achieved by removing the contamination layers. In contrast, solid state lasers require much higher aerial energies to achieve a high bonding strength (>250 mJ/mm^2^). For pre-treatment, the top resin layer has to be completely removed until the fibers are exposed. Unfortunately, at these high intensities and due to the ablation mode, the robustness is relatively small, which results in a high presence of delaminations. For comparable materials and after a treatment with CO_2_-lasers, a high bonding strength is gained starting with aerial energies around 200 mJ/mm^2^. However, due to with this treatment intensity, the risk of thermal degradation is relatively high [9,11,12,13,14].

This leads to the approach within the presented investigations that identifies the crucial aerial energy from which the favorable cohesive failure could be achieved with the new laser source emitting a 3 µm wavelength. Taking this into account, the pre-treatment efficiency in comparison with conventional laser sources was found by pre-treating the samples with the same aerial energy and, subsequently, comparing the obtained bonding strengths rather than varying the aerial energy to achieve for each laser source high bonding strength and, subsequently, compare the differences in terms of the aerial energy.

### 2.2. Laser Systems

In addition to the developed laser system with an output wavelength of 3012 nm, the samples were also treated with conventional lasers. UV-laser a Coherent^®^ AVIA^TM^ 355-23 (Coherent, Inc., Santa Clara, CA, USA) was used. The laser achieves a maximum average power of 23 W depending on the repetition rate (common from 50 kHz to 200 kHz within this examinations set to 200 kHz). The repetition rate depending on the pulse duration was in the range of 30 ns for the present study. Furthermore, a fiber-laser called the SPI-G4 pulsed fiber-laser (SP-070P-AHS-H-C-Y, SPI Lasers, Ltd., Southampton, UK) emitted radiation with a wavelength of 1064 nm and a maximum average output power of 70 W for repetition rates from 55 kHz to 1000 kHz, which was chosen to be 70 kHz (pulse duration is in the range of around 100 ns). In addition, a CO_2_-laser (Diamond E 400, Coherent, Inc.) emitting laser radiation at a wavelength of 10,600 nm was used. This laser has a maximum average output power of 400 W and can operate from a single shot up to 200 kHz of pulse frequency (set to 50 kHz). The pulse length of this laser was around 1.2 µs during this study.

To generate the targeted laser radiation, the output of an industrial solid state IR-laser (CleanLASER CL 150, Clean-Lasersysteme GmbH, Herzogenrath, Germany) had its frequency converted to a wavelength of 3 µm. The industrial laser provides up to 105 W of average power with a pulse duration of 120 ns at pulse frequencies from 11 kHz to 15 kHz (set to 12 kHz). The frequency conversion is implemented as a two-stage scheme comprising an optical parametric oscillator (OPO) and generating the target wavelength and a subsequent optical parametric amplifier (OPA). The frequency-converted laser provides up to 18 W of average power at a wavelength of 3012 nm and a pulse frequency of 12 kHz. A detailed presentation of the setup and the laser characteristics can be found in Reference [19].

For all laser set-ups, the laser beam was focused by an F-Theta optics (resulting in a spot diameter of 300 µm (GEWOL-laser), 200 µm (CO_2_-laser), 95 µm (fiber-laser), and 35 µm (UV-laser)) and guided on the specimen surface by a galvanometer scanner.

### 2.3. Materials and Measurements

The composite samples were fabricated out of a typical aerospace prepreg system (HexPly^®^ 913, HTS fiber from Hexcel Composites GmbH, Stade, Germany), which have a curing temperature of 125 °C. The lay-up was chosen to be [0°/90°/0°/90°/0°]s, which results in a post-cure thickness of roughly 2.5 mm. The composite plates were manufactured in an autoclave with an applied pressure of 7 bar while the vacuum was approximately −0.8 bar compared to the atmosphere. To achieve representative surface contaminations, the aluminum mold was coated with a polysiloxane-based release agent (Chemlease^®^ R&B EZ from Chem-Trend L.P. Howell, MI, USA) and no additional release film was applied.

The demolded CFRP plates were cut with a water-cooled circular saw. After laser pre-treatment with a single scanning process, the specimens were bonded with a typical one component epoxy based adhesive (3M^TM^ Scotch-Weld^TM^ Structural Adhesive Film AF 163-2K from 3M Corp., St. Paul, MN, USA) with a curing temperature of 125 °C and a post-cure thickness of around 0.1 mm, which was guaranteed by an implemented net within the adhesive.

The bonding capability and the correlating efficiency of the bonding pre-treatment was mechanically tested within the single lap shear test following DIN EN 1465 [20] with a slightly different testing rate of 10.0 mm/min and a varied specimen’s width of 20 mm due to the size of the scanning device. The mechanical tests were performed using a universal testing machine Instron 5584 (Instron Deutschland GmbH, Darmstadt, Germany).

The analytical characterization of the surface was completed by using the Fourier transform infrared spectroscopy and a laser scanning microscope. The FTIR measurements were performed with a Bruker Tensor27 (Bruker Corporation, Billerica, MA, USA) working on the basis of attenuated total reflectance (ATR). For the optical judgement of the surface, a Keyence VK-X Series 3D Laser Scanning Confocal Microscope (Keyence Deutschland GmbH, Neu-Isenburg, Germany) was used.

## 3. Results

The main criteria to judge the effectiveness of a pre-treatment method is the achieved bonding strength, which can be correlated with the removal of contamination layers. Therefore, mechanical and analytical tests were performed within this study.

### 3.1. Mechanical Tests

Since the objectives of the study was to evaluate the effectiveness of a 3 µm—laser treatment, examinations were performed to identify a sensitive treatment intensity treatment parameter, which results in high bonding strengths without damaging the composite. Furthermore, the efficiency of the developed laser source can be compared with conventional laser sources by applying the same aerial energy on the sample’s surface and comparing the resulting bonding performance (strength and fracture pattern).

#### 3.1.1. Identification of Pre-Treatment Parameters

**Figure 2:**
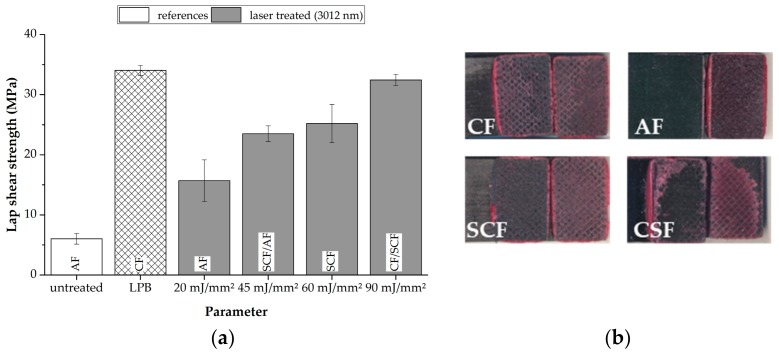
Lap shear results of differently treated samples (**a**) and representative fracture patterns (**b**).

The surfaces of the composite samples were treated with different aerial energies. This was achieved by adapting the scanning parameters. The results of the lap shear tests are shown in Figure 2a while Figure 2b is showing typical fracture patterns of a cohesive failure (CF), an adhesion failure (AF), a substrate close cohesive failure (SCF), and a delamination showing a cohesive substrate failure (CSF).

Focusing the results of the initial tests to identify a sensitive treatment intensity, it can be seen that already low treatment intensities of 20 mJ/mm^2^ lead to a significant increase (more than a factor of 2.5) of the bonding strength compared with the untreated specimens, which only achieve an average lap shear strength of 6.0 ± 0.9 MPa. Nevertheless, the failure mode of these specimens is still an adhesion failure, which leads to the conclusion that higher aerial energies are required to achieve a sufficient cleaning effect. By increasing the aerial energy, the lap shear strength can be increased and the failure mode changes can change to be more and more cohesive inside the adhesive. At an aerial energy of 90 mJ/mm^2^, an average lap shear strength of 32.5 ± 0.9 MPa is achieved. This value is close to the value of the reference treatment method (low pressure blasting), which is 34.0 ± 0.8 MPa. Higher aerial energies do not lead to increased bonding strengths due to the fact that the cohesive performance of the applied adhesive is obtained.

#### 3.1.2. Bonding Performance after Treatment with Radiation of Different Laser Sources

Based on the previously presented results, a treatment with an aerial energy of 90 mJ/mm^2^ was identified to be superior and, therefore, all laser systems were adjusted to apply this aerial energy (see Table 1). This was based on the methodology of this paper for applying the same energy levels and comparing the fracture patterns.

**Figure 3:**
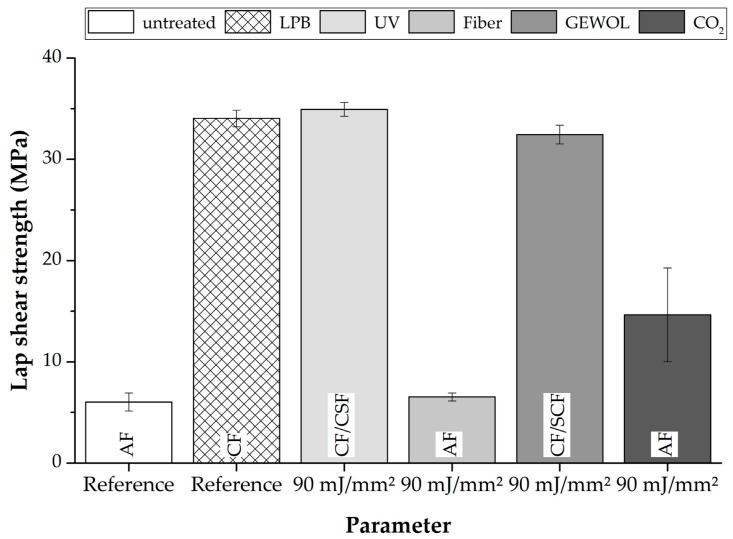
Comparison of the bonding performance of samples treated with different laser sources.

Figure 3 shows the relevant outcome of the surface treatment experiments using the stated laser and treatment parameters represented by the lap shear strength of the bonded. With regards to the achieved strengths, it can be seen that, under a treatment using the UV-laser (355 nm wavelength), the GEWOL-laser (3012 nm wavelength) and low pressure blasting device leads to similar values of lap shear strength. The highest value of 34.9 ± 0.7 MPa is achieved using the UV-Laser. In contrast, the treatment using the fiber-laser (1064 nm wavelength) leads to values of lap shear strength falling far short of the values of the UV-laser. Specimens treated with the fiber-laser fail on a comparable strength level of roughly 6 MPa similar to the untreated reference specimens and also show an adhesion failure. The treatment with the CO_2_-laser at the stated parameters still leads to adhesion failure even though the force level and the correlating lap shear strength rises to 14.6 ± 4.6 MPa.

To fully judge the effectiveness of the treatment, the fracture patterns have to be considered. With regard to the fracture patterns, the UV-laser treatments show small disadvantages due to the fact that, after this treatment, delaminations are present, which may be caused by the slightly reduced matrix absorbance (around 82%) and correlate with higher interaction of the radiation with the carbon fibers. When examining the samples, which were treated with laser radiation of 3012 nm wavelength and by low pressure blasting, no delaminations could be found.

### 3.2. Analytical Measurements

To argue the observed differences in terms of the lap shear strength after different laser treatments, the chemical composition of the surface can be analyzed.

#### 3.2.1. Fundamentals by Analyzing the Reference Specimens

**Figure 4:**
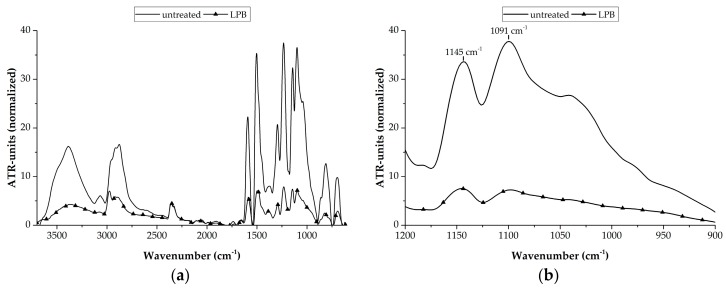
ATR-spectra of the reference specimens: (**a**) global overview, (**b**) focus at characteristic wavenumbers.

Therefore, FTIR-measurements were performed, which are presented in Figure 4 for the reference surface states. It shows an untreated and a low pressure blasted surface.

**Figure 5:**
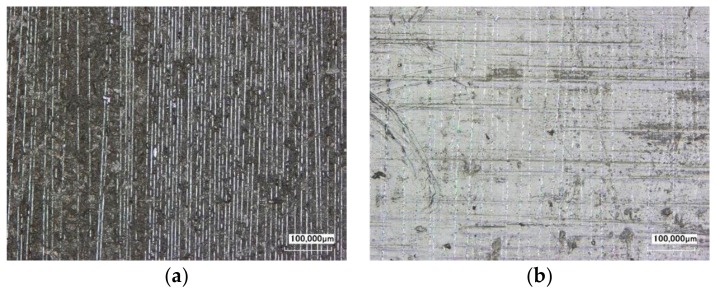
Appearance of the CFRP surface: (**a**) after low pressure blasting and (**b**) untreated.

An overview about the normalized ATR-spectra of the references is given in Figure 4a. It can be seen that, for low pressure blasted samples, all signals are reduced. This happens in the region around a wavenumber of 3000 cm^−1^ as well as in the fingerprint region of the infrared spectrum below 1700 cm^−1^. These effects can be correlated with the occurring surface state. The peaks in the region of 3000 cm^−1^ mainly correlate with CH-bindings and OH-bindings [21], which are part of the chemical composition of the matrix resin and humidity. This is located inside the matrix. Due to this fact, it is plausible that the peaks of the low pressure blasted specimens are less pronounced because the resin layer is significantly removed (see Figure 5b). Due to this reason, the peaks at wavenumbers below 1700 cm^−1^ are less pronounced. These peaks correlate with different organic compounds due to their bindings (as well as signals out of the matrix resin including out of the release agent). A removal of these layers leads to a decreased intensity within this region.

To determine the removal of the contamination layers, a detailed look of the wavenumber region between 1200 cm^−1^ and 900 cm^−1^ is useful. Within this region, the untreated specimens show two significant peaks. Those peaks are located at 1145 cm^−1^ and 1091 cm^−1^ (see Figure 4b). The peak a 1145 cm^−1^ can be correlated with a C—O-binding [22] and the peak at 1091 cm^−1^ is formed by a Si—O-binding [23]. Based on the fact, the C—O-binding is a significant part of an epoxy resin [21] and the Si—O-binding is highly representative for a polysiloxane chain [23]. The peak expression at 1145 cm^−1^ can be correlated with the matrix thickness (which is relatively thick for untreated specimens (Figure 5b) and, by analyzing the ratio of both peaks to itself, the contamination degree can be stated. Therefore, a relative contamination value *C* can be calculated by subtracting the peak height at 1145 cm^−1^ (*E*_1145_) from the peak height at 1091 cm^−1^ (*E*_1091_) (Equation (1)), which is subsequently divided by the 1145 cm^−1^ to take the resin layer thickness into account.

*C* = (*E*_1091_ − *E*_1145_)/*E*_1145_ (1)

Based on this calculation, a clean, contamination free surface is characterized by a small *C* value while a large value correlates with strong contamination. The peak height at a wavenumber of 1145 cm^−1^ is a characteristic value for the resin thickness or the resin share on the surface and the *C* value is an indicator for the surface contamination. Both the peak height and *C* value are shown in Table 2.

Both values can be well correlated with the observed surface state. While the relative high values for the untreated specimens can be linked with a relatively thick resin layer, its present polysiloxane is contaminated. This isn’t the case for the low pressure blasted specimens. The resin share on the surface is low (correlating with a small peak height at 1145 cm^−1^) and the remaining resin does not show any contaminations (very low negative *C* value), which indicates that the values below zero correlate with a silicone-free surface.

#### 3.2.2. Cleaning Effect of Different Laser Sources

**Figure 6:**
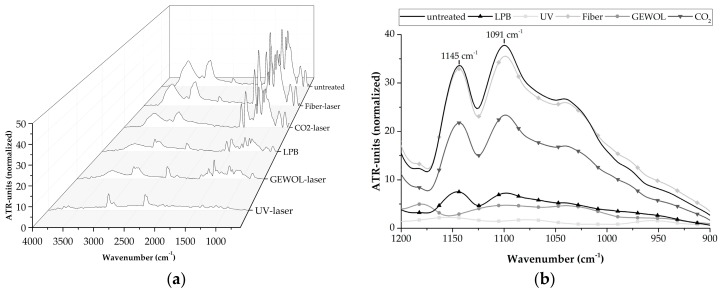
ATR-spectra of the laser treated and reference specimens: (**a**) overview, (**b**) focus at characteristic wavenumbers.

With this knowledge, the cleaning effect of the different laser treatments can be analyzed. Therefore, the global ATR-spectra of the laser and the reference specimens are shown in Figure 6a. The relevant detail of the ATR-spectra is shown in Figure 6b.

Focusing on the overview of the ATR spectra (Figure 6a), there are already significant differences between the different treatment methods. One difference is the reduction of peak impression in the region around the wavenumber of 3000 cm^−1^, which shows high reduction for a treatment with the UV-laser and almost no reduction for the fiber-laser treated samples. Furthermore, there are significant differences between the different treatment methods in the fingerprint region. As mentioned before, these peaks are highly critical for determining the remaining silicone contamination of the CFRP surfaces after the specific pre-treatment.

Therefore, the relevant region within the ATR-spectra is presented in Figure 6b. It can be observed that the expression of the relevant peaks is almost identical when comparing the untreated specimens with treated specimens, which were treated using the fiber-laser. In contrast, both peaks are reduced after treatment with CO_2_ laser radiation despite the fact that they are still well pronounced. This is not the case for the specimens that were treated with laser radiation at 355 nm and at 3012 nm and were low pressure blasted. The ATR-spectra of all of those specimens show a depressed expression in the fingerprint region, the peak at 1145 cm^−1^, and an even more significant depression of the peak at 1091 cm^−1^. This optical impression can be underlined by the relevant, analytical values, which are shown in Table 3.

Comparing the values in Table 3, it can be concluded that there are no contaminations left on the surface after a treatment with 355 nm laser radiation. This is indicated by the high negative *C* value. This is also the case for the specimens, which were treated with laser radiation at 3012 nm wavelength while the *C* value of the surfaces were treated with 1064 nm radiation using the stated treatment parameters, which is relatively high. As mentioned before, the specimens treated with 1064 nm laser radiation show almost the same value as the untreated specimens while the values of the specimens treated with 10,600 nm radiation indicate that there is still a significant resin layer present on the surface. Additionally, a relevant amount of silicone residues remains on the surface.

## 4. Discussion

In general, there is a very satisfactory correlation between the analytical and the mechanical tests. For specimens that were measured to have a low contamination value and a relatively clean surface, high bonding strengths could be achieved. In addition, the depression of other ATR-information (reduction of the fingerprint region and the peaks in the range of 3000 cm^−1^ wavenumbers) can be correlated with an ablation of the matrix resin from the surface, which reduces the peaks generated by the resin itself (fingerprint region) and shares of humidity inside the resin (range around 3000 cm^−1^ wavenumbers).

The results of the mechanical and the analytical measurements can be justified with the ablation mode of the different laser sources and their correlating place of interaction between the laser radiation and the specimens. Therefore, two relevant facts have to be considered. It is the absorption of the laser radiation inside the resin and it is also the photon energy (*E_p_*) of the laser radiation.

Focusing treatment with laser radiation of 1064 nm wavelength, it has to be noticed that the resin is almost fully transparent for this wavelength (see Figure 1). This leads to the fact that, during the pre-treatment of the CFRP specimens, the radiation is transmitted through the top resin layer and is absorbed by the carbon fibers. The applied treatment intensity was kept constant to compare the laser sources by analyzing the bonding performance. The general 90 mJ/mm^2^ were too low to remove the top layer. Therefore, it is not possible to remove the contamination, which results in a surface state that is comparable to the untreated one. This correlates with an inferior bonding performance that results in a subpar efficiency based on the chosen approach. In perspective, an increase in terms of aerial energy would lead to high bonding strength, but, due to this high intensity, the risk of material damage is high and the process window is very small [11,12,13].

In contrast, laser radiation with 355 nm wavelength is fairly good absorbed inside the resin, which results in an ablation mode that can be characterized as partly top-down and partly removed by the difference of thermal expansion of fibers and resin. This leads the top resin layer to be blasted away. In correlation with the high photon energy of about 3.48 eV, it is possible to remove the resin layer from the carbon fibers. This results in a contamination free surface and high bonding capability. However, since the ablation is also (partly) characterized by a thermally based blasting process of the resin, there is a potential of damaging the lower fiber layers, which is underlined by the small shares of delaminations within the fracture patterns of the bonded specimens. With focus on the wavelength of 3012 nm and 10,600 nm in Figure 1, it is obvious that, for both wavelengths, the ablation mode is top-down due to the high absorption inside the resin. Therefore, the differences in terms of the cleaning effect and the correlating bonding capability of the pre-treated specimens have to be explained by the photon energy of the radiation [24,25]. The photon energy can be calculated to 0.41 eV for the 3012 nm and 0.11 eV for the 10,600 nm laser radiation. Based on this, the reduced cleaning effect of the CO_2_ laser radiation can be justified by the fact that a higher treatment intensity is necessary to remove the contamination layer through photo-thermal and multiphoton-based bond breaking [13,26]. Some share of the applied aerial energy is dissipated by heat transfer trough the specimens. This effect is less pronounced for treatment with the radiation emitted by the GEWOL laser.

By summarizing these facts, it can be stated that the introduced state of the art about the laser bonding pre-treatment of composites can be complemented by a surface treatment with laser radiation of 3 µm wavelength. Based on the performed analytical and mechanical tests, the following conclusions can be made. In comparison with conventional laser sources and by applying the same aerial energy on the samples’ surfaces, a treatment with this laser radiation leads to similar bonding strengths than UV-laser radiation without the challenges of using UV-radiation (especially UV-aging of surrounding parts) and the reduced lifetime of UV-laser sources. In addition, the emitted radiation can be guided through a fiber, which results in benefits regarding their applicability within the industrial process chain. Compared with IR-laser radiation (around 1 µm), the radiation with 3 µm wavelength is beneficial due to their high absorbance in the resin layer, which resulted in a more favorable ablation mode (top-down) and, therefore, in a better, more efficient cleaning of the composite surfaces. This is underlined by significantly higher bonding strength when the same treatment intensity (aerial energy) is applied. A better cleaning and correlating bonding performance of the GEWOL-laser can also be observed when compared with a typical CO_2_-laser. Again, when applying the same treatment intensity on the specimen, the bonding performance measured by single lap shear tests is significantly higher. Due to the fact that the resin shows the same high absorbance for both wavelengths, the difference seems to be based on the higher photon energy of the 3 µm radiation, which results in a more efficient ablation mode. Thus, based on the reduced required treatment, the risk to cause material damage leads to delaminations by a thermal over treatment with CO_2_-laser radiation. However, CO_2_-lasers result in a higher process robustness due to treatment with 3 µm laser radiation.

## 5. Conclusions and Outlook

The presented examinations show that laser radiation with a wavelength of 3012 nm can be used for a sufficient bonding pre-treatment of CFRP structures, which is underlined by the analytical measurements regarding the cleaning effect as well as the mechanical bonding tests. In comparison with conventional laser sources (fiber-laser, frequency tripled solid state laser, and CO_2_-laser) a comparable (frequency tripled solid state (UV) laser) or a better cleaning effect resulting in high bonding strengths could be achieved. Based on the presented and previous examinations [8,18], the applicability of the developed laser source could be proven even for the low emitted average output power.

However, to finally establish laser radiation as the preferred method for bonding pre-treatment processes, further work has to be performed. For an industrial application, the laser radiation of 3 µm is promising but the average output power has to be increased to be able to enlarged the spot sizes and increase the pre-treatment speeds, which is comparable with the low pressure blasting process.

In addition, further research should be pursued to investigate the influence of different related materials (especially the potentially present degree of thermal degradation) and the influence of laser source related topics (for example, pulse lengths). This topic leaves relevant room for further research because especially ultra-short pulse laser sources seem promising due to the non-linear absorption and correlating ablation mode of former transparent material [27]. Within this topic, it needs to be investigated whether a specific wavelength (like the present 3012 nm, correlating with a high absorption in the resin) is still more promising than the non-linear ablation mechanisms for ultra-short pulses (i.e., emitted by an IR-laser). Based on these future investigations, laser radiation can be established as a tool for bonding pre-treatment.

## Figures and Tables

**Table 1:** Laser parameters for pre-treatment with different laser sources.

Parameter	UV-Laser	Fiber-Laser	GEWOL-Laser	CO_2_-Laser
Aerial energy (mJ/mm²)	90	89	90	89
Spot diameter (µm)	~35	~100	~300	~200
Average power (W)	12	10	12	13
Scan speed (mm/s)	2700	2800	1220	2660
Repetition rate (kHz)	200	70	12	50
Spot and line overlap (%)	~40	~58	~66	~73
Wavelength (nm)	355	1064	3012	10,600

**Table 2:** Characteristic, analytical parameters for the reference specimens.

Parameter	Untreated	LPB
Peak height at 1145 cm^−1^	32.71	7.52
*C* value	+1.09 × 10^−1^	−0.08 × 10^−1^

**Table 3:** Characteristic, analytical parameters for the laser-treated specimens.

Parameter	UV-Laser	Fiber-Laser	GEWOL-Laser	CO_2_-Laser
Peak height at 1145 cm^−1^	2.24	33.62	4.98	21.78
*C* value	−2.19 × 10^−1^	+1.05 × 10^−1^	−0.46 × 10^−1^	+0.73 × 10^−1^
